# The burden of hip and knee osteoarthritis in Finnish occupational healthcare

**DOI:** 10.1186/s12891-021-04372-9

**Published:** 2021-05-29

**Authors:** Milla Summanen, Liisa Ukkola-Vuoti, Samu Kurki, Samuli Tuominen, Rami Madanat

**Affiliations:** 1 Pfizer Oy, Tietokuja 4, 00330 Helsinki, Finland; 2 Medaffcon Oy, Tietäjäntie 2, 02130 Espoo, Finland; 3 Terveystalo Biobank Finland, Humalistonkatu 7B, 20100 Turku, Finland; 4 Terveystalo Kamppi, Jaakonkatu 3, 00100 Helsinki, Finland

**Keywords:** Osteoarthritis, Occupational healthcare, Registry study, Real world data, Healthcare resource utilization, Burden of disease

## Abstract

**Background:**

Osteoarthritis (OA) is a leading cause of disability and pain especially among older adults, but it is also known to affect working age individuals, often leading to reduced productivity and increased healthcare usage. The aim of this study was to determine the burden of hip and knee OA in Finnish occupational healthcare.

**Methods:**

This was a retrospective registry study utilizing the electronic medical records of the largest private and occupational healthcare provider in Finland. All consented patients with hip or knee OA were identified. A subcohort of occupational healthcare (OCH) patients was then compared to an age- and gender-matched control group without OA. Patient demographics including comorbidities were determined and healthcare contacts, medication prescriptions, and sick leaves were compared between the two groups. The study period was from January 1st, 2012 to April 30th, 2020.

**Results:**

51,068 patients with hip or knee OA were identified (all OA cohort) and 35,109 of these formed the occupational healthcare subcohort. Most of the OA patients were female and belonged to the age group 50–59 years. The point prevalence of hip/knee OA at the end of the study period was 5.6% for the occupational healthcare subcohort. OA patients had 2.2 times more healthcare contacts and 2.8 times more overall sick leave days compared to the age- and gender-matched control cohort. Etoricoxib was the most commonly prescribed medication at OA-related visits (21.8% of patients). Opioids were prescribed to 10.6% of patients at OA-related visits and the most prescribed opioid was a combination of codeine and paracetamol (4.8% of patients). 5054 OA patients (14.4%) had a contraindication for non-steroidal anti-inflammatory drugs (NSAIDs).

**Conclusions:**

This retrospective registry study utilizing real-world data provides new evidence on the disease burden of hip or knee osteoarthritis from the electronic medical records of Finnish occupational healthcare customers. OA patients had more comorbidities, more healthcare contacts, more sick leave days, and more analgesic prescriptions compared to an age- and gender-matched control cohort without OA.

**Supplementary Information:**

The online version contains supplementary material available at 10.1186/s12891-021-04372-9.

## Background

Osteoarthritis (OA) is the most common form of arthritis and a leading cause of disability and pain, especially among older adults [1]. According to the Global Burden of Disease Study, in 2017 there were 303.1 million prevalent cases of hip or knee OA globally, and the age-standardized prevalence rates had increased 9.3% from 1990 to 2017 [2]. Several studies have shown that OA is more prevalent among women and in the oldest age groups [2–4].

However, OA also affects younger, working age individuals, often resulting in reduced productivity and increased healthcare resource utilization [3, 5, 6]. For example, studies utilizing data from the National Health and Wellness Survey in the US found that a substantial proportion of workers suffer from OA pain, and workers with OA have significantly higher healthcare resource utilization and costs compared to those without OA [6, 7]. Further studies have shown that working age adults with OA have a lower employment rate compared to the age group without OA [3], and that OA patients are at an increased risk of work loss due to illness or disability [8, 9].

Even though previous research has clearly demonstrated that OA is a substantial burden also for the working age population, more information is needed on the clinical characteristics, treatment options and healthcare resource utilization of employed OA patients specifically. The aim of this study was to investigate these parameters in patients with either hip or knee OA compared to age- and gender-matched controls, utilizing the electronic medical records (EMRs) of occupational healthcare customers at Terveystalo, which is Finland’s largest private and occupational healthcare provider. Our main hypothesis was that the diagnosis of OA is a significant burden in Finnish occupational healthcare (OCH), demonstrated by the higher use of medical services and more sick leave days recorded for OA patients compared to controls without OA.

## Methods

In Finland, all employees are entitled to preventive occupational healthcare financed and arranged by the employer. Additionally, employers can choose to provide employees with access to medical care, and care coverage at least at the general practitioner level is often included. Electronic medical records (EMRs) of Terveystalo, the largest private and occupational healthcare provider in Finland, were utilized in this retrospective registry-based study. Terveystalo has about 300 clinics around Finland, and in 2019 the total number of medical doctor visits was 3.7 million, or approximately 15% of the nationwide total visits [10]. The study period was from January 1st 2012 until April 30th 2020.

Two OA patient cohorts were formed: the all OA cohort consisting of consented patients with hip or knee OA, and the OCH subcohort from the OCH patients with hip or knee OA. The study inclusion criteria were diagnosis of either hip or knee OA (ICD-10 codes M16* or M17*, respectively), active patient consent available, being adult (≥18 years) at the date of first diagnosis, and health registry data available and accessible. If a patient was diagnosed with both hip and knee OA, the first recorded diagnosis was used for analyses. An additional inclusion criterion for the OCH subcohort was active OCH status. Patients were followed from the first M16/M17 diagnosis (index date) until the end of follow-up, which was defined as the end of the study period (30-Apr-2020), death or end of OCH customership. Separate OCH follow-up periods with a maximum of 3 months in between were combined into a single period. If the break between OCH periods was more than 3 months, only the first continuous follow-up period containing the OA diagnosis was included in the analyses.

In order to determine the burden of hip and knee OA, a one-to-one age (birth year), gender, and length of follow-up matched control cohort of patients without OA was formed from the OCH patients. The controls were followed starting from the index of the corresponding cases. The exclusion criteria for the control cohort were diagnoses of hip, knee, or other OA (ICD-10 codes from M15* to M19*) or arthralgia (M25.5).

The EMRs included in this study were diagnoses, visits, procedures, prescriptions, laboratory results, sick leaves, and demographic characteristics.

### Prevalence

The point prevalence at the end of the study was calculated by dividing the number of prevalent (i.e. alive) and consented OCH patients by the number of prevalent and consented patients recorded at the Terveystalo EMRs on the 30th of April 2020.The point prevalence was reported by gender and type of OA in six age groups.

### Comorbidities and treatment practices

The frequency of comorbidities and co-diagnoses was assessed from the EMRs based on ICD-10 codes (three-character level) overall during the follow-up period. A patient was counted only once even if the same diagnosis code was recorded multiple times. Of special interest were OA-related diagnoses (M15-M19), arthralgia (M25.5), rheumatoid arthritis (M05-M06) and contraindication diagnoses for non-steroidal anti-inflammatory drug (NSAID) use.

OA medication prescriptions for the OCH subcohort were reported separately for OA-related visits and all visits. For controls, prescriptions from all visits were reported. Of special interest were pain medication prescriptions and prescriptions for medications that are contraindications for NSAID use.

An NSAID contraindication was recorded for those patients whose EMRs contained either a diagnosis (ICD-10-code) that is a contraindication for NSAID use, or a prescription for a medication that is a contraindication for NSAID use (or both) according to the national treatment guidelines [11] during the follow-up period (see Additional file 1 for list of diagnoses and medications). Diagnosis codes and prescriptions from all healthcare contacts, not only OA-related contacts, were taken into account here.

### Healthcare resource utilization

For OA patients, healthcare resource utilization (HCRU) consisted of both total and disease-specific healthcare contacts and sick leaves. For disease specific HCRU, an ICD-10 diagnosis code M16* or M17* was required as a diagnosis for the visit/sick leave. The controls had no OA-specific HCRU, so only total HCRU was calculated for them. All healthcare contacts with diagnosis codes (including e.g. physician’s telephone consultation) were included in the calculations for healthcare visits. It should be taken into account that only one primary diagnosis code is recorded for each sick leave period in the Terveystalo EMRs, but it is possible to record multiple diagnosis codes per visit, and for healthcare contacts all recorded diagnosis codes were taken into account in the analysis.

Overlapping records of sick leaves were combined into one continuous sick leave period and counted only once, whereas subsequent, non-overlapping sick leaves were counted individually. Sick leaves beginning prior to the index date or ending after the end of follow-up were included, however, only the sick leave days occurring during the follow-up period were counted.

HCRU data is presented per patient-year (cumulative number of events/cumulative follow-up). Hence, the data is adjusted for differences in follow-up that would otherwise bias those with longer follow-up to more likely have higher HCRU.

### Statistical methods

Data management and statistical analysis were performed using Python 3.7.4 with pandas, spicy stats, and other standard data analysis packages. Only pseudonymized data was used in the analyses to protect patient privacy. The Chi-squared test was used to test for differences in frequencies for categorical variables (comorbidities and prescriptions). The fold-changes between the cases and controls for the comorbidities were calculated by dividing the frequency of cases by the frequency in controls. *P*-values less than 0.05 were considered statistically significant and no multiple testing correction was applied.

## Results

### Study cohort formation and baseline characteristics of the patients

**Fig. 1:**
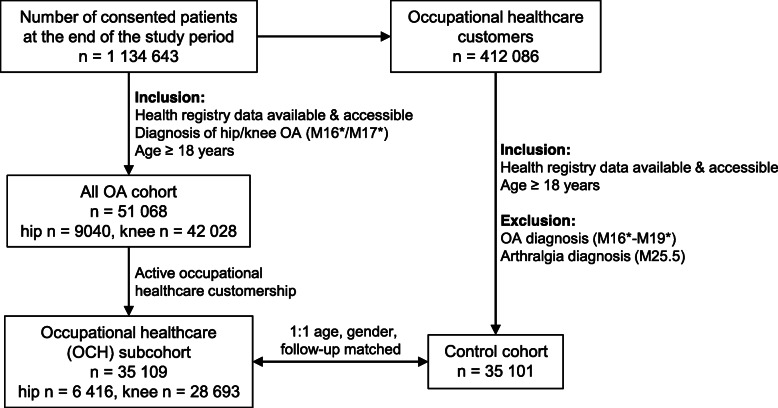
The formation of the study cohorts. The all OA cohort (*n* = 51,068) including adult patients diagnosed with hip or knee OA was formed from the total number of consented patients at the end of the study period. The occupational healthcare (OCH) subcohort (*n* = 35,109) included those OA patients with occupational healthcare customership. The control cohort without OA (*n* = 35,101) was formed from the occupational healthcare customers in the total number of consented patients

The number of consented patients at Terveystalo EMRs at the date of data extraction (30-Apr-2020) was 1,134,643, of whom 412,086 patients were OCH customers (Fig. 1). The all OA patient cohort consisted of 51,068 patients diagnosed with either hip or knee OA (*n* = 9040 hip and *n* = 42,028 knee) during the study period. For the 3025 patients who had both hip and knee OA, the first recorded diagnosis was used for further analyses. An OCH subcohort of 35,109 patients (*n* = 6416 hip and *n* = 28,693 knee) with OCH customership was formed from the all OA cohort. A randomly selected, one-to-one age (birth year), gender and OCH follow-up length matched control cohort of patients without OA was formed for the OCH subcohort. No suitable controls were found for eight elderly OA patients with OCH customership, so the control cohort was eight patients smaller.

Just over half of the patients included in the study were female (Table 1). The age at index was 56.6 years for the all OA cohort and 53.3 years for the OCH subcohort. Most of the patients belonged to the age group 50–59 years. Body mass index (BMI) was available more often for the OCH subcohort than the all OA cohort patients. For those with BMI data available, the mean BMI in the OA cohorts was 30.1 for the hip/knee OA patients, and 27.6 for the patients in the control cohort. Patients in higher BMI groups were seen more often in the OA cohorts, for example, 7.0% of knee OA patients had a BMI higher than 40, compared to only 2.1% of the control cohort patients. Smoking status was only available for between 23.4 and 33.7% of the patients. For those with smoking status available, the majority (68.1 to 74.0%) were non-smokers. The mean follow-up time for the OCH subcohort was 3.4 years (3.4 years for hip and 3.5 years for knee OA patients), and 3.5 years for the control cohort (Table 1).

**Table 1:** Baseline characteristics of the study cohorts

	All OA patients						OCH subcohort						Controls	
	Hip		Knee		Hip/Knee		Hip		Knee		Hip/Knee			
	n/mean	%/SD	n/mean	%/SD	n/mean	%/SD	n/mean	%/SD	n/mean	%/SD	n/mean	%/SD	n/mean	%/SD
Sample size	9040	17.7%	42,028	82.3%	51,608	100%	6416	18.3%	28,693	81.7%	35,109	100%	35,101	100%
Mean follow-up (years)	n/a	n/a	n/a	n/a	n/a	n/a	3.4	2.3	3.5	2.4	3.4	2.4	3.5	2.4
Gender (female)	4902	54.2%	23,849	56.7%	28,751	56.3%	3407	53.1%	15,633	54.4%	19,040	54.2%	19,034	54.2%
Mean age at index	56.8	10.4	56.6	10.1	56.6	10.1	53.3	7.9	53.2	7.6	53.3	7.7	52.4	7.6
Age group at index														
18–29	98	1.1%	328	0.8%	426	0.8%	78	1.2%	271	0.9%	349	1.0%	406	1.2%
30–39	410	4.5%	1845	4.4%	2255	4.4%	369	5.8%	1620	5.6%	1989	5.7%	2170	6.2%
40–49	1687	18.7%	8139	19.4%	9826	19.2%	1528	23.8%	6990	24.4%	8518	24.3%	9703	27.6%
50–59	3902	43.2%	18,795	44.7%	22,697	44.4%	3347	52.2%	15,356	53.5%	18,703	53.3%	18,634	53.1%
60–64	1479	16.4%	6487	15.4%	7966	15.6%	981	15.3%	3961	13.8%	4942	14.1%	3664	10.4%
≥65	1464	16.2%	6434	15.3%	7898	15.5%	113	1.8%	495	1.7%	608	1.7%	524	1.5%
BMI available	4012	44.4%	18,014	42.9%	22,026	43.1%	3799	59.2%	17,134	59.7%	20,933	59.6%	16,400	46.7%
Mean BMI (kg/m^2^)	28.7	5.3	30.4	5.9	30.1	5.9	28.6	5.2	30.4	5.9	30.1	5.8	27.6	4.9
BMI group														
< 25	1001	25.0%	2959	16.4%	3960	18.0%	943	24.8%	2780	16.2%	3723	17.8%	5422	33.1%
25–30	1678	41.8%	6811	37.8%	8489	38.5%	1607	42.3%	6506	38.0%	8113	38.8%	6809	41.5%
30–35	881	22.0%	4778	26.5%	5659	25.7%	833	21.9%	4552	26.6%	5385	25.7%	2918	17.8%
35–40	306	7.6%	2211	12.3%	2517	11.4%	284	7.5%	2093	12.2%	2377	11.4%	905	5.5%
> 40	146	3.6%	1255	7.0%	1401	6.4%	132	3.5%	1203	7.0%	1335	6.4%	346	2.1%
Smoking status available	2126	23.5%	9816	23.4%	11,942	23.4%	2102	32.8%	9656	33.7%	11,758	33.5%	9686	27.6%
Non-smoker	1572	73.9%	7149	72.8%	8721	73.0%	1556	74.0%	7028	72.8%	8584	73.0%	6600	68.1%

Data are shown as the number and percentage of patients, or the mean +/− SD

### Comorbidities and other diagnoses

Comorbidities and other co-diagnoses were assessed from the EMRs based on ICD-10 codes (three-character level) overall during the study follow-up period. The most common comorbidity diagnosis was J06 (ICD-10), acute upper respiratory infection, which was detected in the EMRs of 63.2% of the OCH subcohort patients and 43.8% of the controls (Table 2). M54, dorsalgia was the second most common comorbidity, and it was 1.8-times more common in the OCH subcohort compared to controls. The big difference seen between OA patients and controls for the diagnosis code M25 (Table 2), is explained by the fact that the diagnosis code M25.5, arthralgia, was an exclusion criterion for the control cohort. The prevalence of arthralgia diagnosis in the OCH subcohort was studied in more detail. Of the OCH subcohort patients, 28.9% were diagnosed with arthralgia prior to OA (*n* = 10,155), and the median time from arthralgia diagnosis to OA diagnosis was 0.6 years. Furthermore, 15.9% of the OCH OA patients had the first recorded arthralgia diagnosis after hip/knee OA diagnosis (*n* = 5578).

**Table 2:** Comorbidities and co-diagnoses with a prevalence ≥10% in the hip/knee OA patients

ICD-10	Description	Hip OA		Knee OA		Hip/Knee OA		Controls			
		n	%	n	%	n	%	n	%	***p***-value	fold change
M17	Gonarthrosis [arthrosis of knee]	1006	15.7%	28,693	100%	29,698	84.6%	0	0.0%	–	–
J06	Acute upper respiratory infections of multiple and unspecified sites	3956	61.7%	18,216	63.5%	22,172	63.2%	15,375	43.8%	< 0.001	1.4
M54	Dorsalgia	3682	57.4%	14,866	51.8%	18,548	52.8%	10,308	29.4%	< 0.001	1.8
M25	Other joint disorders	2936	45.8%	13,427	46.8%	16,363	46.6%	277	0.8%	–	–
J01	Acute sinusitis	2099	32.7%	9802	34.2%	11,901	33.9%	7009	20.0%	< 0.001	1.7
J20	Acute bronchitis	1990	31.0%	9663	33.7%	11,653	33.2%	6424	18.3%	< 0.001	1.8
M79	Other soft tissue disorders	2307	36.0%	9160	31.9%	11,467	32.7%	4773	13.6%	< 0.001	2.4
M75	Shoulder lesions	2087	32.5%	9258	32.3%	11,345	32.3%	5602	16.0%	< 0.001	2.0
M23	Internal derangement of knee	679	10.6%	10,452	36.4%	11,131	31.7%	1265	3.6%	< 0.001	8.8
I10	Essential (primary) hypertension	1857	28.9%	9101	31.7%	10,958	31.2%	6969	19.9%	< 0.001	1.6
M53	Other dorsopathies	1583	24.7%	7103	24.8%	8686	24.7%	4515	12.9%	< 0.001	1.9
M77	Other enthesopathies	1361	21.2%	6701	23.4%	8062	23.0%	3379	9.6%	< 0.001	2.4
R10	Abdominal and pelvic pain	1412	22.0%	6091	21.2%	7503	21.4%	4394	12.5%	< 0.001	1.7
M16	Coxarthrosis [arthrosis of hip]	6416	100.0%	1006	3.5%	7493	21.3%	0	0.0%	–	–
S83	Dislocation, sprain and strain of joints and ligaments of knee	499	7.8%	5708	19.9%	6207	17.7%	1461	4.2%	< 0.001	4.2
H10	Conjunctivitis	1034	16.1%	4939	17.2%	5973	17.0%	3580	10.2%	< 0.001	1.7
M70	Soft tissue disorders related to use, overuse and pressure	1566	24.4%	4303	15.0%	5869	16.7%	1874	5.3%	< 0.001	3.1
F51	Nonorganic sleep disorders	998	15.6%	4463	15.6%	5461	15.6%	3551	10.1%	< 0.001	1.5
A09	Diarrhea and gastroenteritis of presumed infectious origin	932	14.5%	4340	15.1%	5272	15.0%	2800	8.0%	< 0.001	1.9
F43	Reaction to severe stress, and adjustment disorders	951	14.8%	4235	14.8%	5186	14.8%	3414	9.7%	< 0.001	1.5
E78	Disorders of lipoprotein metabolism and other lipidaemias	909	14.2%	4114	14.3%	5023	14.3%	3371	9.6%	< 0.001	1.5
N95	Menopausal and other perimenopausal disorders	963	15.0%	4058	14.1%	5021	14.3%	3615	10.3%	< 0.001	1.4
R07	Pain in throat and chest	914	14.2%	3989	13.9%	4903	14.0%	2799	8.0%	< 0.001	1.8
R74	Abnormal serum enzyme levels	807	12.6%	3926	13.7%	4733	13.5%	2832	8.1%	< 0.001	1.7
R05	Cough	823	12.8%	3785	13.2%	4608	13.1%	2609	7.4%	< 0.001	1.8
L30	Other dermatitis	794	12.4%	3507	12.2%	4301	12.3%	2608	7.4%	< 0.001	1.6
M51	Other intervertebral disc disorders	1053	16.4%	3132	10.9%	4185	11.9%	1558	4.4%	< 0.001	2.7
M76	Enthesopathies of lower limb, excluding foot	677	10.6%	2944	10.3%	3621	10.3%	1038	3.0%	< 0.001	3.5
M19	Other arthrosis	650	10.1%	2963	10.3%	3613	10.3%	0	0.0%	–	–
R53	Malaise and fatigue	676	10.5%	2885	10.1%	3561	10.1%	2020	5.8%	< 0.001	1.8
J45	Asthma	594	9.3%	2961	10.3%	3555	10.1%	1898	5.4%	< 0.001	1.9
J02	Acute pharyngitis	621	9.7%	2874	10.0%	3495	10.0%	2006	5.7%	< 0.001	1.7

Data is organized based on prevalence in the hip/knee OA patients. *P*-values and fold changes were calculated between all OA patients and all controls. The *p*-values were calculated using the Chi-squared test. *P*-values and fold changes were not calculated for diagnosis codes, which were exclusion criteria for the control cohort

In addition to the comorbidities shown in Table 2, there were several rarer comorbidities with a prevalence less than 10%, such as type 2 diabetes mellitus (ICD-10 code E11) and chronic obstructive pulmonary disease (COPD; ICD-10 code J44). The prevalence of type 2 diabetes mellitus was 6.5% for hip OA patients, 8.2% for knee OA patients, 7.9% for hip/knee OA patients, and 4.5% for controls (fold change 1.8, *p*-value < 0.001). COPD was also more common in the OA patients than controls, but the overall prevalences were lower (1.4% for hip OA patients, 1.2% for knee OA patients, 1.2% for hip/knee OA patients, and 0.7% for controls; fold change 1.8, *p*-value < 0.001).

### OA prevalence

**Fig. 2:**
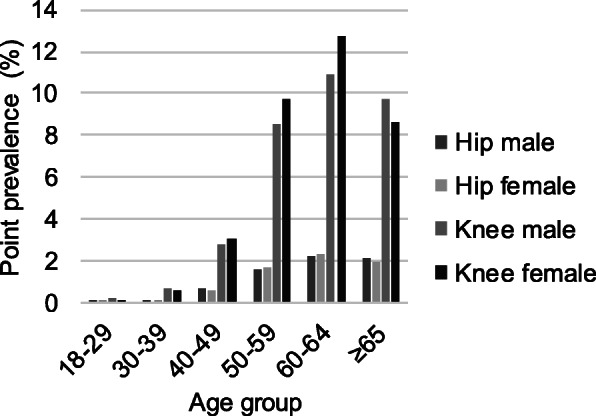
Prevalence of hip/knee OA in the OCH subcohort. The prevalence of hip or knee OA in the OCH subcohort was calculated at the end of the study period (April 2020) and reported by gender, type of OA and age group. The overall point prevalence was 5.6%, and knee OA was 5.5-times more common than hip OA

The point prevalence of hip or knee OA in the OCH subcohort at the end of the study period was 5.6% (5.2% for males and 5.9% for females). Knee OA was 5.5-times more common with a point prevalence of 4.4%, compared to 0.8% for hip OA prevalence. In addition, 1325 patients (0.3%) had both hip and knee OA. The prevalence of OA for both males and females increased with age (Fig. 2). The highest prevalence numbers were seen in the age group 60–64 years, where the prevalences of hip OA were 2.2 and 2.3% for males and females, respectively, and the corresponding numbers for knee OA were 10.9 and 12.8%.

### Current treatment practices

OA medication prescriptions for the OCH subcohort were extracted separately from OA-related visits and all visits to Terveystalo. The most prescribed OA medication was etoricoxib, which was prescribed to 21.8% of OA patients at OA-related visits, and to 52.7% when all visits were taken into account (Table 3). All of the OA medications listed in Table 3 were prescribed to OA patients more often than to controls. The biggest differences between OA patients and controls were seen in the prescriptions for chondroitin sulfate, glucosamine and hyaluronic acid, which were prescribed almost exclusively to OA patients compared to controls (Table 3).

**Table 3:** OA medication prescriptions from OA visits and all visits

ATC code	Medication	OA visits						All visits									
		Hip OA		Knee OA		Hip/Knee OA		Hip OA		Knee OA		Hip/Knee OA		Controls		***p***-value	fold change
		n	%	n	%	n	%	n	%	n	%	n	%	n	%		
M01AH05	etoricoxib	1450	22.6%	6215	21.7%	7665	21.8%	3352	52.2%	15,166	52.9%	18,518	52.7%	6118	17.4%	< 0.001	3.0
M01AE01	ibuprofen	815	12.7%	3741	13.0%	4556	13.0%	3264	50.9%	15,172	52.9%	18,436	52.5%	9741	27.8%	< 0.001	1.9
N02BE01	paracetamol	1228	19.1%	4396	15.3%	5624	16.0%	3233	50.4%	13,749	47.9%	16,982	48.4%	6644	18.9%	< 0.001	2.6
M01AB05	diclofenac	420	6.6%	1671	5.8%	2091	6.0%	1845	28.8%	8152	28.4%	9997	28.5%	4201	12.0%	< 0.001	2.4
M03BX02	tizanidine	190	3.0%	452	1.6%	642	1.8%	1841	28.7%	7846	27.3%	9687	27.6%	5070	14.4%	< 0.001	1.9
M01AX25	chondroitin sulfate	723	11.3%	4137	14.4%	4860	13.8%	1252	19.5%	6841	23.8%	8093	23.1%	352	1.0%	< 0.001	23.0
N02AJ06	codeine & paracetamol	434	6.8%	1255	4.4%	1689	4.8%	1363	21.2%	5498	19.2%	6861	19.5%	2631	7.5%	< 0.001	2.6
M01AE02	naproxen	240	3.7%	987	3.4%	1227	3.5%	1128	17.6%	5297	18.5%	6425	18.3%	2789	8.0%	< 0.001	2.3
N02AA59	codeine, combinations	279	4.4%	819	2.9%	1098	3.1%	1155	18.0%	4835	16.9%	5990	17.1%	2017	5.8%	< 0.001	3.0
M01AX05	glucosamine	447	7.0%	2488	8.7%	2935	8.4%	895	14.0%	4884	17.0%	5779	16.5%	374	1.1%	< 0.001	15.5
M02AA15	diclofenac, topical	112	1.8%	815	2.8%	927	2.6%	801	12.5%	4269	14.9%	5070	14.4%	1624	4.6%	< 0.001	3.1
M01AC06	meloxicam	196	3.1%	1001	3.5%	1197	3.4%	796	12.4%	3662	12.8%	4458	12.7%	1206	3.4%	< 0.001	3.7
M01AE52	naproxen & esomeprazole	234	3.7%	809	2.8%	1043	3.0%	673	10.5%	2955	10.3%	3628	10.3%	1021	2.9%	< 0.001	3.6
N02AX02	tramadol	279	4.4%	669	2.3%	948	2.7%	762	11.9%	2803	9.8%	3565	10.2%	1043	3.0%	< 0.001	3.4
N06AA09	amitriptyline	97	1.5%	305	1.1%	402	1.2%	581	9.1%	2148	7.5%	2729	7.8%	1019	2.9%	< 0.001	2.7
M02AA07	piroxicam	32	0.5%	270	0.9%	302	0.9%	344	5.4%	1880	6.6%	2224	6.3%	733	2.1%	< 0.001	3.0
N03AX16	pregabalin	62	1.0%	171	0.6%	233	0.7%	336	5.2%	1203	4.2%	1539	4.4%	573	1.6%	< 0.001	2.7
M01AH01	celecoxib	90	1.4%	241	0.8%	331	0.9%	237	3.7%	860	3.0%	1097	3.1%	236	0.7%	< 0.001	4.6
N03AX12	gabapentin	49	0.9%	137	0.5%	186	0.5%	259	4.0%	791	2.8%	1050	3.0%	349	1.0%	< 0.001	3.0
M09AX01	hyaluronic acid, intra-articular	29	0.5%	648	2.3%	677	1.9%	53	0.8%	980	3.4%	1033	2.9%	12	0.0%	< 0.001	86.1

Data is organized based on prevalence in the hip/knee OA patients from all visits. *P*-values and fold changes were calculated between all OA patients from all visits and all controls. The *p*-values were calculated using the Chi-squared test

We also looked at the prescription data based on the step-wise approach described in the national treatment guidelines [12], where the first-line pharmaceutical treatment for hip or knee OA is paracetamol, followed by NSAIDs and finally opioids. Nearly half (47.6%) of the patients had no pain medication prescriptions from OA-related visits and 1.9% had a prescription for paracetamol only, and no NSAID or opioid prescriptions. 40.0% had an NSAID prescription but no opioid prescriptions, and the remaining 10.6% had an opioid prescription. The most prescribed opioid was the combination of codeine and paracetamol, which was prescribed to 4.8% of the OA patients at OA-related visits (6.8% of hip OA patients, and 4.4% of knee OA patients; Table 3). When all visits were taken into account, a total of 12,987 OA patients (37.0%) had an opioid prescription in their EMRs, which was 2.5-fold more than was recorded for controls (*n* = 5155; 14.7%).

Hyaluronic acid was prescribed to nearly 3% of OA patients in the OCH subcohort (all visits, Table 3). However, hyaluronic acid is also available as a medical device and these treatments are not seen in the prescription data. Therefore, we also looked at the joint injection procedure codes from the EMRs. The procedure code TNX10, which is used most often for joint injections in Terveystalo EMRs, was recorded for 1994 OA patients (5.7%) in the OCH subcohort (*n* = 1827, 6.4% knee, and *n* = 167, 2.6% hip) compared to only 255 controls (0.7%).

NSAIDs were the most used pain medication for OA patients, but several patients also had NSAID contraindications. In the OCH subcohort, the EMRs of 4026 patients (11.5%) had an ICD-10 code that is a contraindication for NSAID use. Furthermore, 1857 patients (5.3%) had a prescription for a medication that is a contraindication for NSAID use (see Methods for list of diagnosis codes and medications). When these two groups were combined, a total of 5054 OA patients (14.4%) had a contraindication for NSAIDs based on either a diagnosis code or a prescription. An NSAID contraindication was almost twice as common in the OCH subcohort compared to the control cohort, where only 2787 patients (7.9%) had a contraindication for NSAIDs.

### Healthcare resource utilization

**Fig. 3:**
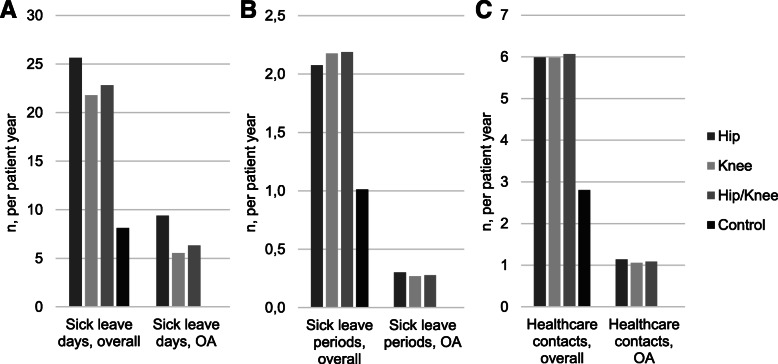
Healthcare resource utilization for the OCH subcohort and controls. The number of sick leave days (**A**), sick leave periods (**B**), and healthcare contacts (**C**) in the OCH subcohort and control cohort per patient year are shown. OA patients had 2.6 times more sick leaves, 2.2 times more sick leave periods and 2.2 times more healthcare contacts compared to controls without OA

OA patients had 2.8 times more overall sick leave days compared to controls (22.8 vs 8.1 days per patient year; Fig. 3A), 2.2 times more sick leave periods (2.2 vs 1.0 per patient year; Fig. 3B), and 2.2 times more healthcare contacts (6.1 vs 2.8 visits per patient year; Fig. 3C). For the OCH OA patients, 28% of all sick leave days were recorded with an OA diagnosis, and 18% of healthcare contacts had OA as one of the visit-related diagnoses. Overall, 78.9% of the OCH OA patients had at least one sick leave day per year, compared to 60% of the controls.

A higher BMI and comorbidities such as type 2 diabetes mellitus and COPD increased healthcare resource utilization for both the OA patients in the OCH subcohort and the controls. For example, hip/knee OA patients with a normal BMI (≤ 25 kg/m^2^) had 16.3 sick leave days and 5.6 healthcare contacts per patient year (PPY), but patients with a BMI > 30 kg/m^2^ had 28.2 sick leave days and 6.8 contacts PPY. The same trend was seen for the controls, but the overall values were lower (7.3 vs 9.4 sick leave days per PPY, and 2.9 vs 3.3 contacts PPY for BMI ≤ 25 kg/m^2^ and BMI > 30 kg/m^2^, respectively). A diagnosis of type 2 diabetes increased the number of sick leave days from 21.7 to 31.2 PPY for hip/knee OA patients and from 7.9 to 12.8 PPY for controls, and the number of healthcare contacts from 5.8 to 7.5 PPY for hip/knee OA patients and from 2.7 to 4.1 PPY for controls. Furthermore, both hip/knee OA patients and controls with COPD had more sick leave days and healthcare contacts per patient year compared to those without this comorbidity (39.0 vs 22.3 sick leave days and 8.2 vs 6.0 contacts PPY for hip/knee OA patients, and 20.3 vs 8.1 sick leave days and 4.8 vs 2.8 contacts PPY for controls).

We also looked at the number of healthcare contacts per patient year by type of professional visited. The type of professional visited most often by both OCH OA patients and controls was an occupational healthcare physician (9.1 visits per patient year for OCH OA patients vs 4.6 for controls). Orthopedic specialists were the most visited specialists for OCH OA patients (total of 53,049 visits, or 0.4 per patient year, compared to *n* = 9753, or 0.1 per patient year for controls).

## Discussion

Even though the prevalence of OA is known to increase with age, hip and knee OA are a significant burden also for the working age population [3, 5, 7]. In this retrospective registry study conducted in the data lake of Finland’s largest private and occupational healthcare provider, the overall prevalence of hip or knee OA in the occupational healthcare subcohort was 5.6% at the end of the study period. As expected, the prevalence increased with age, peaking in the age group 60–64 years. In a recent study with data from the United States, the prevalence of OA in the age group 50–64 years was 14.8% [3], which is very similar to the 12.5% seen in this study for the same age group. The prevalences reported here are also comparable to the latest Finnish prevalence data from 20 years ago, where the prevalence of hip or knee OA in the age group 55–64 years was 14.4% for males and 11.2% for females [13].

In contrast to the other studies mentioned above, which reported even higher prevalences in the age groups over 65 years, in this study the prevalence of OA was lower in the age group 65 years and older compared to 60–64 years (12.6% vs 15.4%). This can be readily explained by the fact that in Finland the most common retirement age is around 65 years, and very few people are covered by occupational healthcare after they turn 65. Therefore, most of the OA patients in the older age groups receive treatment for their OA from the public healthcare sector and other data sources would be needed to access that data. Since data for age groups older than 65 years is not comprehensive in the data source used for this study, we also did not report the OA prevalence for the all OA cohort.

Importantly, our study shows that hip or knee OA diagnosis increases HCRU, as demonstrated by the increase in both sick leave days and healthcare contacts compared to controls without OA. Out of the 22.8 sick leave days per patient year recorded for OA patients (vs. 8.1 days for controls), 6.3 days were caused by OA specifically. Therefore, comorbid conditions were responsible for 8.4 days of the increase seen for OA patients. A similar pattern was seen for healthcare contacts, with 1.1 of the 6.1 healthcare contacts per patient year due to OA specifically. In general, HCRU was similar for hip and knee OA patients, although patients with hip OA had slightly more sick leave days compared to patients with knee OA (25.6 vs 21.8 sick leave days per patient year). Other studies have also described substantial increases in days missed from work due to OA. For example, Kotlarz et al. reported that OA increased annual absenteeism from 5.5 to 9.2 days for women and from 5.2 to 9.7 days for men [5], and Sharif et al. showed that OA cases had a 90% higher hazard ratio of work loss due to illness or disability compared to non-OA controls over a 2 year period [8].

On average, the OA patients had a higher BMI and more comorbidities recorded in their EMRs compared to the age- and gender-matched controls without OA. For example, dorsalgia was 1.8 times more common in the OA patients. Other studies have also reported higher BMIs [3, 7] and a higher comorbidity burden [7, 14] for patients with OA compared to those without. Furthermore, 14.4% of the OA patients in this study had a contraindication for NSAID use based on either diagnosis codes and/or prescription data. This data supports the findings from previous studies and highlights the need for a multidisciplinary approach in the treatment of OA, and the importance of considering possible comorbid diagnoses and other prescriptions.

OA treatment guidelines, including the Finnish national guidelines, emphasize the role of non-pharmacological interventions, rehabilitation, and exercise programs in the treatment of OA [12, 15, 16]. According to the national treatment guidelines [12], pharmaceutical treatment for OA pain should be initiated with paracetamol, followed by NSAIDs, whereas opioids are seen as the last resort. Based on these guidelines, the number of paracetamol prescriptions reported here (16.0% on OA-related visits) is quite low. However, paracetamol is also available over the counter from Finnish pharmacies, and patients may also have a prescription from the public healthcare sector, which is not seen in this data. Furthermore, it should be noted that while 16.0% of OA patients had a paracetamol prescription, it was the only analgesic prescription for only 1.9% of the patients, indicating that most of these patients had an NSAID/opioid prescription as well.

Based on recent literature from other European countries, it seems that around one third of OA patients have NSAID prescriptions or use NSAIDs for OA pain. For example, Spitaels et al. reported that in Belgium 29.4% of knee OA patients had an oral NSAID prescription and 2.3% had a prescription for a cyclooxygenase-2 (COX-2) selective NSAID [17]. In a Dutch study, 30% of hip/knee OA patients used NSAIDs with diclofenac being the most commonly used drug in this group (15%) [18]. In our study, NSAIDs were prescribed to 40% of OA patients in the OCH subcohort, and the COX-2 selective NSAID etoricoxib was the single most prescribed analgesic (21.8% on OA-related visits).

Even though opioid use is seen as the last resort in the national treatment guidelines, opioid prescriptions were relatively common, with 10.6% of patients having an opioid prescription from an OA-related visit. The vast majority of the opioid prescriptions reported here were for weak opioids, including tramadol. The Dutch and Belgian studies reported very similar values, with opioid use recorded for 12% of OA patients in the Netherlands [18], and weak and strong opioid prescriptions to 6.1 and 4.3% of OA patients, respectively, in Belgium [17].

Looking at prescriptions from all visits, not just those with an OA-diagnosis, 37.0% of the OA patients in the OCH subcohort had an opioid prescription, which is 2.5 times higher compared to the controls. This is comparable to a recent study from Sweden, where the 12 month prevalence of opioid use among hip/knee OA patients was 23.7%, and 2.1-fold higher compared to those without hip/knee OA [19]. In a follow-up study, Thorlund et al. reported that more than half of the incident opioid dispensations to hip/knee OA patients within the first year after diagnosis were inappropriate according to current treatment guidelines [20]. Considering the updated treatment guidelines and the results from a recent meta-analysis, which concluded that opioids provide minimal relief for OA symptoms and cause discomfort in a majority of patients [21], it is important to pay close attention to the pattern of opioid prescriptions for patients with hip or knee OA.

To our knowledge, this the first real-world data study focusing specifically on employed, working age individuals diagnosed with either hip or knee OA. The data source used in the study, the Terveystalo data lake, provides a geographically comprehensive data set, and with 1.1 million consented patients covers up to 21% of the Finnish population. As occupational healthcare must be provided by the employer according to Finnish law, there is also no selection of patients due to societal status or position. Additional strengths include the fact that data was collected in a real-world setting without stringent inclusion and exclusion criteria, and the fact that diagnoses, prescriptions, sick leave days and visits can all be accessed from the same data source.

However, like all real-world data studies, this study also has some limitations. Some information may not have been consistently recorded for all patients, potentially affecting the study population and other outcomes. The data source does not contain data on drug purchases, so the use of medication in daily dose equivalents cannot be determined. Furthermore, the data source does not currently contain any patient reported outcome measures (PROMs), such as pain or physical function measures or patient global assessments, which would be relevant for OA patients [22, 23]. Such measures would also be required to truly measure the value of healthcare [24]. With the increasing prevalence of OA due largely to an aging population and the increasing prevalence of obesity [2, 4], we expect the OA-associated HCRU to increase correspondingly. This will form a challenge for the healthcare system and highlight the need to build value-based care pathways for patients suffering from chronic diseases such as OA, requiring the implementation of PROMs into clinical practice.

## Conclusions

This retrospective registry study utilizing real-world data provides new evidence on the disease burden of hip or knee osteoarthritis from the EMRs of Finnish occupational healthcare customers. The OA patients had more comorbidities, more analgesic prescriptions, more sick leave days, and more healthcare contacts compared to an age- and gender-matched control cohort without OA. Importantly, this study demonstrated that even though NSAIDs are the most used analgesic for OA pain, up to 14% of OA patients have a contraindication for NSAID use based on the EMRs. Furthermore, although opioids are seen as the last resort for OA treatment according to the updated national and international treatment guidelines, almost 11% of OA patients in this cohort had an opioid prescription from an OA-related visit. These results highlight the need for novel therapies for the safe management of chronic pain associated with OA [25]. Moreover, this study clearly shows that effective and value-based OA treatments are needed, especially considering the increasing prevalence of this chronic condition.

## Supplementary Information

**Additional file 1.** NSAID contraindication diagnoses and prescriptions. The table includes the diagnoses codes and prescriptions that were used to determine NSAID contraindications for the study cohorts.

## Data Availability

The datasets generated and analyzed during the current study are not publicly available due to restrictions in the Finnish legislation but are available for research purposes from Terveystalo (visit https://www.terveystalo.com/fi/Yritystietoa/Terveystalo-Biopankki/Biopankki/Tietopyynto/ or email biopankki@terveystalo.com for more information) subject to approval by the appropriate data permit authority.

## Abbreviations

OA: Osteoarthritis
NSAID: Non-steroidal anti-inflammatory drug
EMR: Electronic Medical Record
OCH: Occupational healthcare
HCRU: Healthcare resource utilization
BMI: Body mass index
COPD: Chronic obstructive pulmonary disease
PPY: Per patient year
COX-2: Cyclooxygenase-2
